# Chemical Characterization and Bioaccessibility Assessment of Bioactive Compounds from Umbu (*Spondias tuberosa* A.) Fruit Peel and Pulp Flours

**DOI:** 10.3390/foods10112597

**Published:** 2021-10-27

**Authors:** Laís B. Cangussu, Pãmella Fronza, Adriana S. Franca, Leandro S. Oliveira

**Affiliations:** 1 PPGCA, Universidade Federal de Minas Gerais, Av. Antônio Carlos, 6627, Belo Horizonte 31270-901, MG, Brazil; lai.sbc1@hotmail.com (L.B.C.); pamellafronza@hotmail.com (P.F.); leandro@demec.ufmg.br (L.S.O.); 2 DEMEC, Universidade Federal de Minas Gerais, Av. Antônio Carlos, 6627, Belo Horizonte 31270-901, MG, Brazil

**Keywords:** trigonelline, technological properties, fruit peels, digestion simulation, non-extractable phenolics

## Abstract

Umbu, a common fruit from the northeastern region of Brazil, contains many bioactive compounds not yet exploited. Thus, this study evaluated the potential of pulps and peels of mature and semi-mature umbu as a source of bioactive compounds. Trigonelline contents ranged from 1.75 to 6.14 mg/100 g, values higher than those of many vegetables described in the literature, such as corn and barley. The contents of extractable and non-extractable phenolic compounds were also higher than those of other vegetables. Bioaccessibility of total extractable phenolics, flavonoids, and tannins was determined (15.67–37.73%, 31.87–39.10% and 18.81–114.27%, respectively). The constituent polysaccharides of the pulp and peel were tentatively chemically characterized as arabinoxylans, arabinogalactans, rhamnoarabinogalactans, xyloglucans, and pectin of the rhamnogalacturonan type. The technological potential of peel flours was evaluated. The maturation advancement showed no significant changes in the technological properties of the flours, except for color and water solubility index. Results indicated excellent prospects for future research on umbu pulps and peels as potential sources of natural bioactive compounds.

## 1. Introduction

Umbu (*Spondias tuberosa* A.) is a tropical fruit typically of semi-arid regions of the Brazilian northeastern region, occurring from Piauí to the north of Minas Gerais State, with significant local economic relevance [1,2]. Its tree, a xerophytic species, forms tubers able of storing water, minerals, and organic solutes. Therefore, it is suitable for arid to semi-arid regions where water is scarce, and the soils are poor [3]. The 2017 production of umbu in Brazil amounted to 7465 tonnes [4]. It has been gaining space in the national and international markets due to its pleasant flavor, peculiar aroma and content of bioactive compounds, such as phenolics [5]. However, many bioactive compounds have not yet been investigated in umbu.

Unlike most plant species, *S. tuberosa* produces fruits in the drought season. Thus, this plant is important during this season for pollinators and dispersers, and their fruits can be an alternative for population consumption, farmers survival, and production of several products: juice [6], mixed drinks [7], cereal bars [8], among others. Therefore, *S. tuberosa* has economic, social, cultural, and ecological importance [9]. Besides, the umbu products industry generates thousands of tonnes of pulp, peels and seeds as by-products [10], which could be further utilized.

Exotic fruits by-products are promising food ingredients in commercial products due to their high contents of bioactive compounds [11]. There are several studies of the incorporation of vegetable wastes fibers into foodstuffs by turning them into powders [12,13]. Powders of vegetable wastes have excellent technological properties such as oil and water retention capacity, high antioxidant capacity and high dietary fibers contents [10,14].

A handful of works have studied the composition of umbu pulp but these studies were restricted to quantifying major classes of compounds, such as total carotenoids and total phenolics [6,15]. There were no studies that quantified the separate phenolic classes (flavonoids, tannins, and phenolic acids) or their individual compounds and that studied the bioaccessibility of these phenolic compounds. Also, there were no studies in which the non-extractable phenolics were quantified and in which the polysaccharides were characterized. A saponification step is usually carried out for the release of esterified carotenoids [16]. However, this aspect was not tackled in the available literature on umbu. Other bioactive compounds widely found in plants are alkaloids, such as trigonelline. However, to the authors knowledge, there are no studies in the literature in which trigonelline was analyzed in umbu pulp and peel. Trigonelline presents bioactive activities, including dental cavities prevention, anti-carcinogenicity [17], and antidiabetic effect [18]. Thus, it was the aim of this study to chemically characterize and quantify the individual compounds in the carotenoids and phenolics classes as well as bioactive compounds not yet exploited, such as polysaccharides and trigonelline, both for pulp and peel, for distinct maturity stages. Also, non-extractable phenolics were quantified. Non-extractable phenolics are polymeric phenolic compounds bound to plant tissue macromolecules that yield bioavailable metabolites by the gut microbiota with significant local and/or systemic effects after absorption. The in vitro bioaccessibility of the phenolic compounds was herein determined. Furthermore, the fruit’s pulp and peel indigestible fractions were characterized, and the potential use of peels flours as an ingredient in food formulations was evaluated by determining their respective technological properties.

## 2. Materials and Methods

### 2.1. Materials

Mature and semi-mature umbu fruits (*Spondias tuberose* A.) were collected in Porteirinha, at the Caatinga biome in Minas Gerais State, Brazil. The fruits were selected by manual and visual inspection. The development stage (maturity) was determined by inspection of the color and texture of the fruits. Mature fruits were characterized by a yellowish hue and soft texture, whereas semi-mature fruits were characterized by a greenish hue and firm texture. Subsequently, the fruits were washed and sanitized with sodium hypochlorite (0.8%), pat dried and stored at −18 °C. The standards used for the HPLC analysis were lycopene ≥ 95%, β-carotene ≥ 95%, lutein ≥ 95%, α-tocopherol ≥ 95%, α-carotene ≥ 95%, zeaxanthin ≥ 95%, β-cryptoxanthin ≥ 95%, retinol ≥ 95%, syringic acid ≥ 95%, 2,5-dihydroxybenzoic acid min. 98%, 3-hydroxybenzoic acid 99%, benzoic acid ≥ 99.9%, 4-hydroxybenzoic acid 99%, quercetin ≥ 95%, quinic acid min. 98%, gallic acid min. 98%, hydroxybenzoic acid 99%, ethyl gallate ≥ 96%, *p*-coumaric acid ≥ 98%, ferulic acid ≥ 99%, chlorogenic acid min. 95%, caffeic acid ≥ 98%, 89 procyanidin B2 ≥ 90%, catechin ≥ 99%, epicatechin ≥ 90% and ellagic acid ≥ 95% from Sigma-Aldrich (Brazil); and protocatechuic acid ≥ 90% from USP (Brazil).

### 2.2. Flour Preparation

The pulps and peels fractions were removed from the thawed fruits and cut into pieces of 4 × 4 × 4 cm. All samples were dried at 60 °C in a convective oven (model 420-1DE, Nova Ética, São Paulo, Brazil) to bring the moisture content to 9% [14]. Dried samples were ground and sieved (355-µm mesh). The resulting flours were termed MPU and MPE, in reference to flours from mature umbu pulp and peel, respectively, and SMPU and SMPE, in reference to pulp and peel of semi-mature fruits, respectively. The prepared flours were then stored at −18 °C in sealed plastic bags until further analyses.

### 2.3. Indigestible Fraction Characterization

Indigestible fraction (IF) is herein defined as complex substances, with significant variations in physical and chemical properties, which are not hydrolyzed by endogenous enzymes in the small intestine, thus reaching the colon, where they are fermented by the local microflora, potentially promoting physiological effects in the human body. Therefore, in this definition, differently than the concept of dietary fibers, indigestible food constituents other than non-starch polysaccharides and lignin, such as indigestible proteins and phenolics, are not excluded. Indigestible fractions were determined by enzymatic-gravimetric method in accordance with Leão et al. [14]. Enzymatic digestions were carried out with α-amylase (15 min, 100 °C), pepsin (pH 1.5, 60 min, 40 °C) and pancreatin (pH 6.8, 60 min, 40 °C). Subsequently, the pH was adjusted for 4.5 and the insoluble indigestible fraction (IIF) was filtered and washed twice with acidulated water. The filtrate and the wash water were mixed with ethanol at 60 °C and let rest for one night to precipitate the soluble indigestible fraction (SIF). SIF and IIF were then washed with ethanol and acetone, dried at 105 °C and incinerated at 550 °C. SIF and IIF masses were obtained by

(1) $$F=\frac{D1-I1-B1}{w}\times 100$$

where *F* = soluble or insoluble indigestible fraction, *D*1 = weight after dried at 105 °C, *I*1 = weight after incinerated at 550 °C, *B*1 = (*D*1 − *I*1) of white crucible and *w* = sample weight.

Pectin content was determined according to the method optimized by Leão et al. [19]. 15 mL of distilled water and 15 mL of citric acid solution (pH 2.0) were added to 0.5 g of sample in a microwave reactor (Synth model Microwave Synthesis Labstation) connected to a condenser to avoid losses by ebullition. The system was heated at 108 °C and 600 W for 3 min with magnetic stirring. After 3 min, the sample was removed from the reactor, filtered (60 Mesh) while still warm, and the pectin gel obtained was immersed in absolute ethylic alcohol, filtered again, dehydrated with acetone and dried at 40 °C in convective oven (Model 420-IDE, Nova Ética, São Paulo, Brazil).

The determination of the monosaccharide composition was performed by the alditol acetate method [20]. The hydrolysis of glycosidic bonds of holocellulosic polysaccharides was carried out for 5 mg samples with 0.5 mL of trifluoracetic acid (TFA) 2 M at 120 °C, for 60 min, and, subsequently, with 125 µL sulphuric acid 72% for 3 h, at 30 °C, for hydrolysis of cellulosic glycosidic bonds. The monosaccharides obtained thereof were reduced to their alditol acetates using sodium borohydride 0.5 M in 1 mL dimethylsulfoxide for 90 min, at 40 °C. The alditol acetylation was performed with 200 µL of 1-methylimidazole and 2 mL of acetic anhydride for 10 min. Dichloromethane (1 mL) was used to extract the alditol acetates, which were analyzed in a Varian 3900 gas chromatograph with a flame ionization detector at 250 °C, injector CP-1177 at 230 °C, a 0.5 split, and a column BPX-70 (30 m × 0.32 mm and 0.25 µm; SGE Chromatography Products). Nitrogen was used as carrier gas (1.5 mL/min). The execution time was of 38 min (38 °C for 30 s, increase of 50 °C/min up to 170 °C, increase of 2 °C/min up to 230 °C, 230 °C for 5 min). The identification of monosaccharides was by individual standards and the relative molar ratio (RMR) for each monosaccharide was obtained by the internal standard allose.

Mid-infrared spectra were obtained for the prepared flours with a Shimadzu IRAffinity-1 Spectrophotometer (Shimadzu, Japan) with a DLATGS (Deuterated Triglycine Sulphate Doped with L-Alanine) detector in the range 650–4000 cm^−1^, at 4 cm^−1^ resolution, and 20 scans. Diffuse reflectance (DR) measurements were performed using a Shimadzu sampling accessory (DRS8000A).

### 2.4. Determination of Bioactive Compounds

Extracts of extractable phenolics were prepared by the procedure described in the literature [21], with modifications. Umbu flours (0.5 g) were extracted in an ultrasonic bath (30 min) with methanol (50% *v*/*v*) and acetone (70% *v*/*v*), 20 mL each. After each extraction, the samples were centrifuged (3500 rpm for 10 min). Afterwards, the supernatants were combined, and the volume was completed with water distilled to 50 mL. Prepared extracts were used to quantify total extractable phenolics, flavonoids, and tannins.

Extractable phenolics (TEP) content was determined by the Folin-Ciocalteau essay [14]. In 1 mL of extract, 1 mL of Folin-Ciocalteu reagent (1:3) and 2 mL of sodium carbonate solution (20% *v*/*v*) were added. The solution was stirred and let to rest. After 90 min, absorbance was measured in a UV/Vis spectrophotometer at 765 nm. TEP was quantified by calibration curve of gallic acid (10 to 80 µg/mL) and the results expressed as gallic acid equivalents (mg GAE per g of dry matter).

Tannins also were quantified by the Folin-Ciocalteu method, before and after insoluble matrix (poly(vinylpyrrolidone)- PVPP) addition [22]. In summary, 100 mg of PVPP and 1 mL of distilled water were added in 1 mL of extractable-phenolics extracts. The tubes were kept at 4 °C for 15 min, centrifuged (3500 rpm, 15 min), and the supernatant was collected. This supernatant contained no tannins, since PVPP binds to tannins. Absorbance was read in a UV/VIS spectrophotometer at 725 nm by the Folin-Ciocalteu method. The total tannin content was quantified by the difference of total phenolics (reading without PVPP addition) and simple phenols (reading after PVPP addition). A calibration curve was built employing tannic acid (10 to 80 µg/mL) and the results were expressed as tannic acid equivalents (mg TAE per 100 g of dry matter). The total flavonoid content was quantified by an aluminum chloride assay [15]. In summary, 1 mL of extract was combined with 0.5 mL aluminum chloride solution (5% *m*/*v*) and 1 mL of methanol. The solution was mixed well, kept resting for 30 min and absorbance was read (425 nm) using a UV/VIS spectrophotometer. Quantification was done by a calibration curve of quercetin and the results were expressed as quercetin equivalents (mg QCE per 100 g of dry matter). The phenolic acids content was calculated as the difference between total extractable phenolics, and the sum of flavonoids and tannins.

Non-extractable phenolics (condensed tannins and hydrolyzable tannins) were evaluated using the residues obtained from the extractable-phenolics extraction. Condensed tannins (proanthocyanidins, NEPA) were determined according to the methodology developed by Zurita et al. [23], in which 10 mL of HCl/butanol (5:95, *v*/*v*) containing 0.7 g of FeCl_3_/L were added to the residues and the mixture kept in a water bath at 100 °C for 1 h. The samples were centrifuged at 3500 rpm for 10 min and supernatants collected. After two washings with HCl/butanol/FeCl_3_ (5 mL each), the volume was completed to 25 mL. Absorbances (450 and 555 nm) were read and the sum of absorbances was used to obtain NEPA concentration. Standard curve was built using polymeric proanthocyanidin concentrate isolated from carob pod (*Ceratonia siliqua* L.).

Hydrolyzable phenolics were extracted and hydrolyzed by methanol/H_2_SO_4_ (90:10 *v*/*v*) at 85 °C for 20 h. The remaining material was filtered, the supernatants collected, and volume completed to 25 mL with distilled water [21]. The total hydrolyzable tannins was analyzed by Folin-Ciocalteu assay [24], similar to that used for total extractable phenolics.

Hydrolyzable tannins are comprised of ellagitannins and gallotannins. Gallotannins content was quantified by the rhodanine method [25]. In 1 mL of hydrolyzable tannins extracts, 1.5 mL of methanolic rhodanine solution 0.67% (*m*/*v*) was added and let to rest for 5 min. Subsequently, 1 mL of 0.5 N aqueous KOH solution and 5 mL of distilled water were added. Five min later, absorbance at 520 nm was read. A calibration curve of gallic acid (10 to 80 µg/mL) was built, and the results were expressed as gallic acid equivalents (mg GAE per 100 g of dry matter). Ellagitannins were obtained by difference.

Trigonelline extraction was based on the method described by Servillo et al. [26]. In summary, 5 mL of boiling Milli-Q water were added to the samples (0.1 g). The tubes were incubated in a Dubnoff bath at 100 °C with shaking for 15 min, subsequently centrifuged at 3500 rpm for 10 min and the supernatant collected. The volume was completed to 5 mL and the extracts filtered through a 0.45 µm syringe filter (PTFE). Extracts (5 μL) were injected in a high-performance liquid chromatograph (Shimadzu Corporation, Japan) with a photodiode array detector and C18 column (Shimadzu 150 mm × 4.6 μm, 5 μm particle size). The analysis was performed at 40 °C with a flow of 0.6 mL/min and a gradient elution of (A) acetonitrile:water:phosphoric acid (2.6:7:0.4) and (B) methanol: 100% A in 0 min, 0% A in 15 min, 100% A in 20 min. Identification of trigonelline was carried out by its retention time and UV-spectra comparison with that of the trigonelline standard. Quantification was based on a calibration curve (area obtained by LabSolutions Copyright-Shimadzu Corporation versus trigonelline standard, 0.5–15.0 μg/mL).

The carotenoids content was determined according to the literature [27,28]. The viability of the saponification execution was evaluated by reading after each stage: extraction, partition, and saponification. Partition is essential when saponification is performed to separate the KOH-methanol solution from the solution with the carotenoids (Rodríguez-Amaya, 2001). Cooled acetone was added to flours (0.5 g:5 mL) and kept resting for 30 min in the dark. The samples were centrifuged, the supernatants filtered and washed twice with 2.5 mL of cooled acetone. The volume was completed to 10 mL with refrigerated acetone. Acetone extracts were transferred to a separatory funnel and 10 mL of petroleum ether (cooled) were added. Four washes with distilled water (approximately 50 mL) were performed to remove acetone. The acetone and water (lower phase) were discarded, and the petroleum ether with the carotenoids was transferred to a volumetric flask. The volume was completed to 10 mL with refrigerated petroleum ether. In the samples with petroleum ether, 10 mL of methanol KOH solution (10% *m*/*v*) were added. Nitrogen was bubbled for a few seconds and the samples kept resting overnight for lipids and chlorophylls saponification. After standing, the samples were transferred to a separatory funnel and 5 mL of petroleum ether was added. The ether was collected, and the methanol discarded. Washes were carried out with distilled water until neutral pH. The ether with the carotenoids was transferred to a volumetric flask and the volume completed to 10 mL with refrigerated petroleum ether. In all steps, the absorbances at 449 nm (zeaxanthin) and 450 nm (β-carotene) were read using a UV/VIS spectrophotometer. Quantification was carried out by:

(2) $$Total\;carotenoids\left(\frac{\mathrm{mg}}{100\;g}\right)=\frac{Abs\times Vf\times 1,000,000}{A_{1\mathrm{cm}}^{1\%}\times ma\times 100}$$

where *Vf* is the final sample volume, *ma* is the sample mass, $A_{1\mathrm{cm}}^{1\%}$ is 2348 and 2340 for zeaxanthin in petroleum ether and in acetone, respectively, and $A_{1\mathrm{cm}}^{1\%}$ is 2592 (petroleum ether and acetone) for β-carotene.

The carotenoids and phenolics profiles were determined according to Cândido et al. [29] and Suzuki et al. [30], respectively, using the high-performance liquid chromatography (HPLC) on a Prominence Model (Shimadzu) using a Photodiode Array Detector (PDA) and a Shimadzu column C18 (4.6 μm × 150 mm). Carotenoids were extracted without saponification since this procedure was not feasible for umbu samples. Identification of specific compounds was carried out by analyzing the UV spectra obtained and comparing the retention time of each compound with the available standards.

### 2.5. Bioaccessibility of Phenolics

The in vitro digestion procedure was accomplished in three phases (oral, gastric, and small intestinal) as reported by Dutra et al. [31], with small modifications. 12.5 mL saline solution (0.05 g/mL of Na_2_HPO_4_, 0.004 g/mL of KH_2_PO_4_, 0.16 g/mL of NaCl and 0.17 g of α-amylase) were added to the samples in amber bottles. The solutions were shaken in an orbital incubator (95 rpm) at 37 °C for 10 min. Subsequently, the mixtures were acidified to pH 2.5 with HCl 3M, and 5 mL of porcine pepsin solution (13 mg of pepsin in 5 mL of 0.1 mol/L HCl) were added. To simulate gastric digestion, the samples were shaken (95 rpm) at 37 °C for 1 h. Subsequently, the samples were immediately cooled in an ice bath. The pH was adjusted to 7.5 with NaHCO_3_ (1M) and 5 mL of 1M NaHCO_3_ containing 87 mg of pancreatin and 7 mg of bile salts were added. The samples were shaken (95 rpm) at 37 °C for 2 h to simulate the small intestinal digestion step. The solutions were centrifuged at 3500 rpm for 10 min and the supernatants collected. The volume was completed to 50 mL with distilled water. The extracts were used to quantify total free phenolics, flavonoids, and tannins. The methodologies were the same used for the extracts without digestion. The bioaccessibility percentage (% *Bio*) was calculated by

(3) $$\%\;Bio=\frac{b\times 100}{a}$$

where *b* is the quantity of phenolics after in vitro digestion and *a* is the quantity of phenolics prior to in vitro digestion.

### 2.6. Proximal Composition and Technological Properties

Proximal composition and technological properties were determined for the prepared flour to evaluate the potential use of peels flours as an ingredient in food formulations. The proximal composition of the flours was determined by AOAC methods [32]. Moisture content was obtained after drying in a convective oven (105 °C) until constant weight. Ash was determined by burning at 550 °C for 20 h (AOAC method 942.05). Soxhlet method (AOAC method 4.5.05) was used for crude fat. Petroleum ether was used for extraction. Crude protein was determined by the Kjeldahl method (AOAC method 960.52). Carbohydrate content was obtained by difference.

Color was measured by a tristimulus colorimeter (ColorFlex, Hunter Lab, Reston, VA, USA) (D65 and 10°). Luminosity L*, parameter a*, and b* were measured and c* (chroma) and h (angle) were calculated (Resende et al., 2019). Swelling capacity (*SWC*), water retention capacity (*WRC*), oil retention capacity (*ORC*) and water solubility index (*WSI*) were evaluated according to Leão et al. [14] and Resende et al. [20] by Equations (4)–(7), respectively.

(4) $$SWC=\frac{volume\;occupied\;by\;hydrated\;sample}{mass\;of\;dry\;sample\;\left(g\right)}$$

(5) $$WRC=\frac{mass\;of\;sample\;with\;water\;\left(g\right)}{mass\;of\;dry\;sample\;\left(g\right)}$$

(6) $$ORC=\frac{mass\;of\;sample\;with\;oil\;\left(g\right)}{mass\;of\;dry\;sample\;\left(g\right)}$$

(7) $$WSI=\frac{mass\;of\;sample\;after\;drying\;\left(g\right)}{mass\;of\;sample\;\left(g\right)}\times 100$$

### 2.7. Statistical Analysis

The determinations were done in two repetitions and triplicates and data expressed as mean values ± standard deviation. The normality of the data was verified by the Shapiro-Wilk method. Data were statistically analyzed using ANOVA and Tuckey tests, with 95% confidence (*p* < 0.05).

## 3. Results

### 3.1. Indigestible Fraction Characterization

The soluble and insoluble indigestible fractions and pectin compositions of the herein-prepared powders of umbu pulps and peels are presented in Table 1. All samples presented higher values of insoluble IF than of soluble IF, similar to the results determined by Ribeiro et al. [5] for umbu pulp (12.35%) and peel (49.34%). The physiological effects associated with insoluble IF are certainly similar to those of dietary fibers, since dietary fibers are included within the indigestible fractions, and include the ability to increase fecal volume and decrease intestinal transit [14]. Peel samples presented total indigestible fraction values significantly higher than pulp samples, being classified as fiber-rich powders [33]. The botanic functions of peel and pulp justified the fiber distribution. The peel has a protection function against external agents and is thus more fibrous than the pulp. The pulp protects the seed and can be fibrous depending on the fruit. Despite presenting lower values than IIF, SIF contents in peels samples were higher than those for pequi (~9.7%), buriti (0.86–3.17%) and kiwi (dietary fiber content of 6.9–9.9%) by-products [14,20,34]. The soluble indigestible fractions are also of functional and technological interest. Thus, our results show that umbu peels have potential as an ingredient in new formulations aiming for enriched food.

Conventional pectin extraction methods are time-consuming and/or use strong mineral acids. To replace the use of these acids and minimize the extraction time, in this study, pectin was obtained using citric acid in a microwave reactor. No studies were found of pectin in umbu pulp and by-products powders under these conditions. The pectin contents herein determined were higher than those determined for soluble indigestible fractions, a result similar to pequi by-products [19]. The soluble fraction of the indigestible fraction certainly is mostly comprised of pectin. However, the extraction of all the pectin content by the methods employed to determine the soluble and insoluble fraction of the indigestible fraction are far from efficient in that regard, i.e., not all the pectin present in a plant tissue is extracted by these methods. Recall that these methods are somewhat mimicking digestibility in the human tract and are not aiming at maximum extraction of specific food constituents. The method employed for the exclusive determination of pectin is optimized for such a task and thus it can certainly give results for pectin contents higher than those for soluble indigestible fractions. Since conventional pectin extraction methods are time-consuming and/or use strong mineral acids, to replace the use of these acids and minimize the extraction time, an efficient microwave-assisted extraction procedure was used in our study. Hence, the pectin contents determined in our study were higher than those determined for the soluble indigestible fraction, an indicative of the efficiency of the pectin extraction method assisted by microwaves.

The samples of semi-mature umbu presented higher contents of pectin than the samples of mature umbu, and the peel samples presented higher contents than the pulp samples due to fruit peels having a greater amount of soluble indigestible fractions than pulps. Seixas et al. [35] used the same microwave conditions to extract pectin from passion fruit peel powders but used tartaric acid instead of citric acid. The pectin content determined in their study (16%) was similar to that of this study for mature umbu peel. Since passion fruit peels are known to be rich in pectin, it can be said that umbu has great potential as a source of pectin and that the method used in this study performed well.

The monosaccharides compositions of the umbu by-products are presented in Table 2. All samples showed high levels of glucose with a higher proportion in the H_2_SO_4_-hydrolyzable polysaccharides fraction, which is expected since cellulose is a biopolymer comprised solely of glucose units and is not hydrolyzable by TFA. On the other hand, the hemicelluloses are hydrolyzable both by TFA and H_2_SO_4_ and, therefore, monosaccharides such as arabinose, xylose, and galactose are present in both TFA- and H_2_SO_4_-hydrolyzable fractions [19]. Mannose was present only in the H_2_SO_4_-hydrolyzable fractions, suggesting it comprises β-mannans. In the TFA-hydrolyzable fraction, the presence of glucose is due to polysaccharides other than cellulose, such glucose-containing hemicelluloses and pectic polysaccharides. The determined contents of glucose, galactose, arabinose, xylose, rhamnose and traces of fucose might be related to the presence of polysaccharides such as arabinogalactans, arabinoxylans and xyloglucans. Regardless of which of the cited polysaccharides are present in the analyzed samples, they are surely present in greater amounts in the peel fraction since these samples presented higher contents of arabinose, galactose, xylose, fucose, and rhamnose than the pulp samples. However, pulp samples showed higher glucose contents. This result indicates that these samples have higher contents of sucrose, which is consistent with the sweetness they present [20].

**Figure 1:**
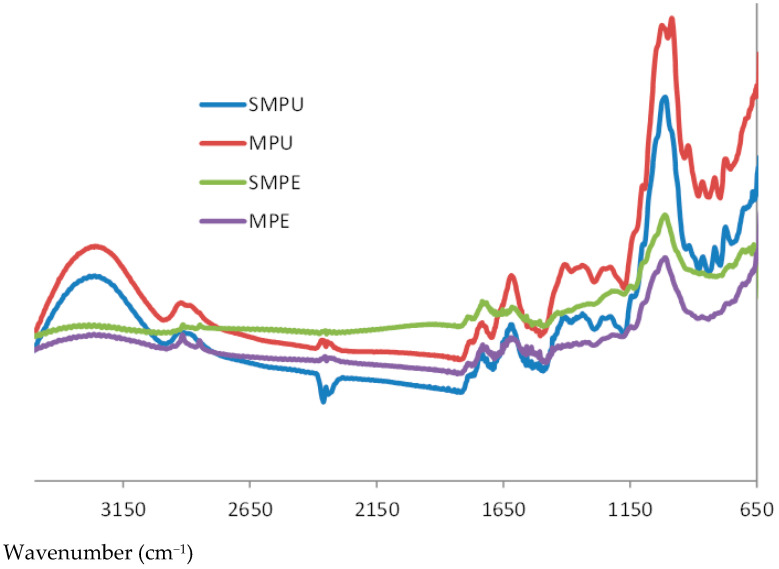
FTIR spectra for umbu peels and pulps. MPE: mature peel; SMPE: semi-mature peel; MPU: mature pulp; SMPU: semi-mature pulp.

The FTIR spectra of umbu peels and pulps (Figure 1) and the absolute values of negative peaks of their second and fourth derivatives were evaluated in the fingerprint region. The wavenumber range of 800–1800 cm^−1^ are of special interest since it is a characteristic range of wavenumbers of polysaccharides that constitute plant cell walls. However, in this region, bands related to amide I (1750–1600 cm^−1^) and II (1500–1400 cm^−1^), associated with proteins [36], can also be found. Thus, the peaks are not well resolved in this portion of the spectra. The wavelength range of 950 and 1200 cm^−1^ is termed the “fingerprint region” for carbohydrates since it allows the identification of their main functional chemical groups and linkages [37]. Due to intensive overlapping of absorption peaks in this region, the second derivative of the spectra was taken, allowing for better resolution of the peaks. The fourth derivative was also taken since there were still a few overlapping peaks in the second derivative.

In all analyzed spectra, bands were observed in the region of 1740–1600 cm^−1^, characteristic of esterified and non-esterified groups in pectins, with the clear band at 1740 cm^−1^ being attributed to carbonyl esters and the band at 1630 cm^−1^ attributed to antisymmetric carboxylates [38]. Bands with maxima at 1259 and 1078 cm^−1^ were observed, with 1259 cm^−1^ being attributed to stretching of C-O bonds in the C-OH groups and 1078 cm^−1^ assigned to stretching of C-C bonds in the main structure of rhamnogalacturonans [39,40,41]. There is also a strong O-CH_3_ band between 3000 and 2800 cm^−1^ due to the methyl ester groups of the galacturonic acid in the pectins. All these results confirm the presence of pectin in umbu samples.

The absorption bands at 1078 and 891 cm^−1^ are characteristic of arabinogalactans type II, with 1078 and 891 cm^−1^ bands being attributed to galactopyranosyl backbone and to stretching vibration of anomeric C-H of β-galactopyranosyl units in arabinogalactan backbone, respectively [40,42]. The presence of peaks at 1078, 1049, 1040 and 891 cm^−1^ indicate the presence of xyloglucans and arabinoxylans in the samples, with wavenumbers 1040 cm^−1^ and 891 cm^−1^ attributed to xylans [43] and the stretching of β-anomeric link in xyloglucans [44]. Specifically, the band at 891 cm^−1^ can also be attributed to C1-H bending of xylose-containing hemicellulose [41]. Absorption bands characteristic of cellulose (in the vicinity of 1200, 1160, 1052, 1030, 988 and 886 cm^−1^) were detected at rather low intensities, visible only in the second derivative of the spectra. Thus, it is fair to infer that hemicellulosic polysaccharides are present in higher amounts than cellulose in umbu pulp and peel samples. Absorption bands characteristic of low-substituted mannans (in the vicinity of 1065, 1027, 1013, 870 and 807 cm^−1^) [41] were not detected in all the spectra, corroborating the results for the GC analysis, which showed small amounts of mannose in the H_2_SO_4_-hydrolyzable fractions. Hence, from the presented analysis, it can be safely inferred that arabinoxylans, arabinogalactans, rhamnoarabinogalactans and xyloglucans are the most probable polysaccharides present in the hemicellulose fractions, aside from pectin of the rhamnogalacturonan type.

### 3.2. Bioactive Compounds

The results for extractable phenolics (Table 3) show that these compounds decreased with the advancement of maturation, as observed in other studies, such as those found by Amira et al. [45]. The peels powders presented the highest values of extractable phenolics. This result can be associated with the fact that many phenolics have the function of defending the plants against external aggressors such as bacteria and insects [14]. The pulp samples presented higher phenolic acid values, while the peel samples presented higher values of tannins, which is consistent with tannins being usually more expressive in the peels than in the pulps of fruits [46].

The pulps samples presented higher bioaccessibility of total extractable phenolics and tannins than the peels samples, with the larger values corresponding to tannins. These results suggest that tannins were more easily removed from the pulps than the peels. Peels and pulps have different cell wall compositions, and this could contribute to promoting changes in gastrointestinal digestion. The peel cell walls were tougher to break than those in the pulp under the gastrointestinal simulation conditions. The samples herein studied presented higher bioaccessibility of total extractable phenolics than those determined for green (5.85%) and mature (2%) carob [47]. The bioaccessibilities of flavonoids in umbu pulps and peels flours (~32–39%) were higher than those for other Brazilian cerrado fruits such as seriguela (12.5%), mangaba (24.95%) and umbu-caja pulp (20%) [31]. Studies of tannins bioaccessibility were not found in the literature.

The results herein obtained for TEP were lower than those determined by Omena et al. [1] for peel and pulp of umbu (5250 and 4040 mg GAE/100 g, respectively). Besides, the result for umbu pulp in our study was higher than those for umbu pulp (15.8 mg EAG/100 g) determined by Zielinski et al. [15] and for umbu juice (193.13 mg EAG/100 g) determined by Ribeiro et al. [6]. Variation in phenolic contents in the same type of fruit is common since several factors influence its final quantity, such as maturation, species, cultivation practices, geographical origin, harvest and storage conditions, among others [48]. The herein determined contents of flavonoids in umbu peels were lower than those of acerola residues and higher than those of jamelão, pitanga, and umbu-cajá, all fruits of the Brazilian cerrado regions [49]. Pulp samples presented flavonoids contents higher than those of papaya, cajá, graviola, pineapple, cashew, cocoa, seriguela, and tamarind fruits and lower than those for grape and açaí [15]. Fruits that presented higher contents of flavonoids than umbu pulps and peels were acerola residues, grape pulp, and açai pulp. The colors of these matrices, corroborating these results since they are blue, reddish-blue, or violet due to the presence of flavonoids.

Non-extractable phenolics (NEPA) contents are shown in Table 3. These are phenolics that are not extracted with aqueous-organic solvents, usually remaining in the residues and not often determined. However, it is important to determine these compounds, since they have been deemed promoters of beneficial health effects such as preventing colorectal cancer and CVD [50]. Non-extractable phenolics, which are macromolecular antioxidants, include high molecular weight polyphenols, such as condensed tannins and low molecular weight polyphenols, associated with proteins and fibers, the hydrolyzable tannins [24].

The highest contents of condensed and hydrolyzable tannins were determined for the peel samples, similar to extractable phenolics. Samples of mature umbu presented higher values of hydrolyzable tannins and lower of condensed tannins than semi-mature samples. The herein studied samples presented higher contents of NEPA in comparison to apple (44.77 mg/100 g), peach (59.1 mg/110 g), nectarine (44.8 mg/100 g) [21] and pequi by-products [14]. These samples also presented higher contents of hydrolyzable tannins in comparison to apple (263.3 mg/100 g), mango (323.7 mg/100 g), nectarine (338.7 mg/100 g), orange (466.8 mg/100 g), and watermelon pulp (520.6 mg/100 g) [24]. No published studies were found on the contents of condensed and hydrolyzable tannins in umbu fruit.

The hydrolyzable tannins are divided into gallotannins and ellagitannins. To the best of our knowledge, there are no published studies in which the contents of these compounds in residues obtained from extractable phenolics were determined. Peels samples presented the highest results of gallotannins and ellagitannins. All samples presented higher ellagitannins contents than those of gallotannins. The ellagitannins are much more common in nature than the gallotannins, being the main tannins present in plant species [51]. After hydrolysis, ellagitannins produce ellagic acids [52], which have beneficial effects in the treatment of liver and Parkinson’s disease [53].

**Figure 2:**
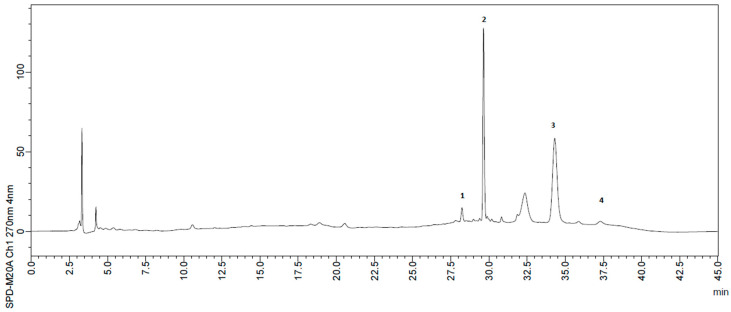
Typical chromatogram for phenolics: 1. *p*-coumaric acid; 2. ellagic acid; 3. Quercetin; 4. procyanidin b2.

The phenolic compounds identified in the samples by HPLC are *p*-coumaric, quercetin, ellagic acid, procyanidin B2, syringic acid and protocatechuic acid (Table 4). A typical chromatogram is presented in Figure 2. We herein emphasize that this is a typical chromatogram and thus is not representative of the relative amounts of each of the compounds therein since the chromatogram was selected for a specific wavelength, which obviously do not correspond to each and every maximum wavelength (λmax) of the compounds therein. Recall that the HPLC detector used was the Photodiode Array Detector (PDA) that sweeps from wavelengths of 180 to 700 nm and is able to provide chromatograms at any specific wavelength in this range. Thus, not all the detected compounds are visible in all specific wavelength chromatograms. Ellagic acid and quercetin were the major phenolics in the peels samples. In contrast, quercetin was not detected in the pulps samples, which also presented ellagic acid as the major phenolic. The phenolic profile found in our study is consistent with that described in a study on umbu plant leaves [54], while some phenolic compounds herein detected (e.g., syringic acid, quercetin and *p*-coumaric acid) were previously reported for umbu pulp [55]. Syringic acid was only detected in SMPE sample at 1.56 ± 0.54 mg/100 g. To the best of our knowledge, this is the first report on the phenolic profile in the umbu peels.

HPLC-PAD analyses indicated that all flours contained trigonelline. Pulp samples presented higher contents (6.14 ± 0.05 and 2.85 ± 1.00 mg/100 g for MPU and SMPU, respectively) than peel samples (3.26 ± 0.18 and 1.75 ± 0.21 mg/100 g for MPE and SMPE, respectively) in the same stage of maturation, similar to those determined by Farag et al. [56] for date fruits. Servillo et al. [57] showed the potential of citrus fruit juices as a source of trigonelline: orange (0.68 mg/100 g), lemon (0.83 mg/100 g), tangerine (0.76 mg/100 g) and grape (0.16 mg/100 g). Mature umbu samples presented higher contents of trigonelline than the semi-mature umbu samples. According to Evans and Tramontano [58], during plant development, trigonelline is synthesized in leaves and is translocated to seeds during fruit maturation, which justifies our results. Umbu pulps and peels flours presented higher contents of trigonelline than rice (1.2 mg/100 g), sorghum (1.14 mg/100 g), corn (1.4 mg/100 g) and barley (1.6 mg/100 g) [26]. Thus, this result demonstrates the bioactive potential of umbu pulp and peel.

The results for carotenoid contents of the samples at each analytical step (Table 5) showed a significant loss of carotenoids in the partition and saponification stages. The partition step caused a reduction of approximately 50% of the carotenoid content and the saponification step a loss of more than 50%. These results demonstrated that it is not feasible to carry out the saponification of the umbu carotenoids since their degradation was more significant than their release. Saponification also eliminates chlorophylls, which may interfere with the analysis. However, the reduction in partition shows that the losses are more related to analysis than chlorophyll interference since in this step there is still no chlorophyll elimination.

Mature pulp flour presented higher contents of carotenoids than the semi-mature pulp. Carotenoids are synthesized mostly during fruit ripening. However, semi-mature umbu peels without saponification presented higher contents of carotenoids than mature umbu peels, different than the results following the saponification stage. This result is justified by the fact that umbu peel is green, indicating the presence of chlorophyll. Thus, determinations without saponification have interferences of chlorophyll, since these compounds were not eliminated. Chlorophyll content of the peel decreases with maturation advancement, with color of the peel changing to yellow. The mature peels presented behavior similar to that of the pulps after the saponification stage. Peel samples had higher values of carotenoids than the pulps samples, similar to the behavior of phenolic compounds. Values herein obtained are high in comparison to buriti by-products [20], kiwi residues [34], strawberry, and graviola [15], with the same extraction conditions. These results confirm the potential of umbu powders as a bioactive ingredient for food formulations.

**Figure 3:**
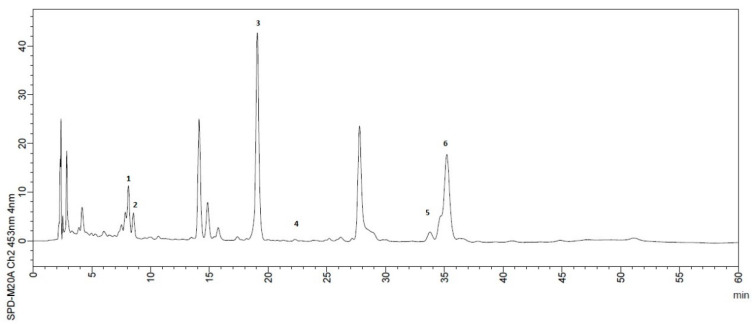
Typical chromatogram for carotenoids: 1. Lutein; 2. Zeaxanthin; 3. beta-cryptoxanthin; 4. alfa-tocopherol; 5. alfa-carotene; 6. beta-carotene.

The carotenoids profile (Table 6) demonstrates that β-cryptoxanthin and β-carotene are the major carotenoids in the samples, similar to found in umbu juice [6] and in seriguela pulp [59]. Dias et al. [60] reported β-carotene, β-cryptoxanthin, and lutein in almost all tropical and subtropical fruits listed, while lycopene was reported as uncommon carotenes in fruits. Thus, it is consistent that lycopene was not found in the samples herein studied. Lutein was the only carotenoid that showed no interference in the total carotenoids differences between samples of peels and pulps and between mature and semi-mature samples. Fat-soluble vitamins were also analyzed. Retinol was not found in the samples. However, α-tocopherol was identified in all samples with major contents in peels samples. These results confirm the potential of these materials valorization. A typical chromatogram is presented in Figure 3. The same comments for the phenolics chromatogram apply herein.

### 3.3. Proximal Composition and Technological Properties

The proximal compositions of the herein-prepared powders of umbu peels are presented in Table 7. Moisture contents were lower than the limit recommended for commercial powders (9 g/100 g) [33]. Low water content is essential to the conservation of the flours to avoid chemical reactions and microorganism proliferation [14]. Thus, the costs of packaging, logistics and chemical preservation methods are reduced. Fat and protein contents did not differ among the samples and were similar to levels reported for buriti peel flours [20], that contain levels as expected to commercial by-products flours. Ash content of the semi-mature samples was lower than those for the mature ones. This result is consistent with the results obtained for date palm fruit [61]. The ash contents are slightly lower than that of yellow passion fruit seeds [10], similar to levels determined for pequi peel [14] and buriti by-products [20], but higher than that of seriguela by-products [12], all of them fruits of the Brazilian semi-arid regions. Ash content can influence the color of the final product but can also be a source of minerals. Carbohydrate content was high, which is expected for fruit peels. The expectation is that the main content of carbohydrate is fibers, which have several health benefits such as intestinal regulation and stool volume [14].

Color parameter results are displayed in Table 8. Luminosity (L*) results are higher than those for pequi peels powders (45.2 < L* > 55.2) [14], passion seed (L* = 43.89) [10] and buriti by-products (53.13 < L* > 62.38) [20], showing that umbu peels powders are lighter than other by-products powders. Lighter powders are desired since they do not interfere with the characteristic color of food. Increased umbu maturation resulted in decreased luminosity of the peels samples due to fruits darkening as maturation proceeds [62]. The samples also provided significant differences in color tone, but all samples presented a yellowish hue.

The technological properties of the produced flours are also displayed in Table 8. Water retention capacity (WRC), oil retention capacity (ORC), and swelling capacity (SWC) did not differ among samples. The water retention capacity (WRC) was high in comparison to buriti residues [20] and yellow passion fruit seeds [10]. SWC values were higher than those for mango and guava residues [63]. Water absorption and swelling volume are important for many products because water interferes with texture and juiciness. Besides, high WRC prevents water loss during cooking. The oil retention capacity is low in comparison to those of coconut residues [64] and pomegranate seeds [65]. However, this result is positive since the low ORC does not cause a caloric increase in the product after exposure to oils. Mature peel samples presented higher values of water solubility index (WSI) than semi-mature samples, which can be explained by mature fruits having higher contents of simple sugars than semi-mature ones. However, both presented higher values of WSI than buriti peels [20]. Low solubility in water is not desirable as it restricts the application in various food products, such as jams. These results confirm that the flours of umbu pulp and peel (by-products of the fruit processing) can be used in food products to replace other flours with lower nutritional and functional values.

## 4. Conclusions

In this study, bioactive compounds not yet explored were duly characterized and quantified in umbu by-product’s flours such as trigonelline, gallotannins, and polysaccharides. High levels of extractable and non-extractable phenolics in umbu pulps and peels flours were found. Besides, peels samples showed greater total indigestible fractions, pectin, phenolic compounds and carotenoids contents than pulps samples, but lower phenolics bioaccessibility and trigonelline contents. All samples indicated presence of pectic polysaccharides and hemicelluloses. It was determined that the saponification step in the quantification of carotenoids was not feasible. The mature peel flour samples presented higher water solubility, being a characteristic determinant of its application. In general, technological, and chemical properties of the by-product’s flours demonstrated that they have potential as sources of bioactive compounds for food applications. In vivo bioaccessibility tests were not carried out in this study, which restrict the analysis of the herein obtained bioaccessibility results to the potential availability of phenolics for absorption. Further studies are being currently conducted to confirm the herein performed tentative identification of the polysaccharides by gas chromatography coupled to mass spectrometry and nuclear magnetic resonance.

## Figures and Tables

**Table 1:** Indigestible fraction characterization of umbu by-products.

Sample	SIF (%)	IIF (%)	TIF (%)	Pectin (%)
MPU	4.58 ± 0.79 ^a^	10.19 ± 1.18 ^a^	14.77	8.73 ± 0.38 ^a^
SMPU	4.30 ± 0.95 ^a^	9.87 ± 0.63 ^a^	14.17	14.57 ± 0.33 ^b^
MPE	13.78 ± 0.51 ^b^	41.26 ± 2.29 ^b^	55.04	16.69 ± 0.27 ^c^
SMPE	13.85 ± 1.33 ^b^	39.71 ± 2.39 ^b^	53.56	20.41 ± 0.06 ^d^

Mean ± standard deviation (*n* = 3). Different letters in the same column indicate that values are significantly different (*p* > 0.05). MPE: mature peel; SMPE: semi-mature peel; MPU: mature pulp; SMPU: semi-mature pulp; SIF: Soluble Indigestible Fraction; IIF: Insoluble Indigestible Fraction; TIF: Total Indigestible Fraction.

**Table 2:** Monosaccharide composition of umbu by-products.

TFA-Hydrolyzable Polysaccharides				
Monosaccharide (%mol)				
Rhamnose	MPU	SMPU	MPE	SMPE
Fucose	0.48 ± 0.10 ^c^	0.48 ± 0.08 ^c^	2.46 ± 0.20 ^a^	1.74 ± 0.07 ^b^
Ribose	0.26 ± 0.04 ^c^	0.28 ± 0.05 ^c^	1.10 ± 0.08 ^a^	0.75 ± 0.05 ^b^
Arabinose	0.38 ± 0.10 ^c^	0.56 ± 0.09 ^c^	2.17 ± 0.23 ^a^	1.34 ± 0.21 ^b^
Xylose	7.40 ± 0.31 ^d^	7.98 ± 0.39 ^d^	18.20 ± 2.29 ^a,b^	21.29 ± 1.39 ^a^
Galactose	1.43 ± 0.1 ^b^	1.26 ± 0.06 ^b^	3.58 ± 0.41 ^a^	3.55 ± 0.11 ^a^
Glucose	5.82 ± 0.49 ^c^	5.95 ± 0.13 ^c^	7.45 ± 0.30 ^b^	14.31 ± 0.15 ^a^
Myo-Inositol	82.55 ± 0.39 ^b^	83.07 ± 0.30 ^b^	63.67 ± 2.04 ^c^	56.66 ± 0.03 ^d^
2-deoxyglucose	0.49 ± 0.02 ^b^	0.41 ± 0.05 ^b^	0.33 ± 0.10 ^b^	0.42 ± 0.11 ^b^
Mannose	0.92 ± 0.08 ^a^	-	1.19 ± 0.39 ^a^	0.95 ± 0.40 ^a^
	-	-	-	-
	H_2_SO_4_-Hydrolyzable Polysaccharides			
Monosaccharide (%mol)	MPU	SMPU	MPE	SMPE
Rhamnose	-	-	-	-
Fucose	-	-	-	-
Ribose	-	-	-	-
Arabinose	6.79 ± 0.88 ^b^	6.56 ± 0.54 ^b^	14.61 ± 0.93 ^c^	16.73 ± 0.91 ^b,c^
Xylose	0.91 ± 0.08 ^c^	0.89 ± 0.26 ^c^	3.11 ± 0.20 ^a^	3.25 ± 0.22 ^a^
Galactose	2.64 ± 0.77 ^a^	3.27 ± 0.19 ^d^	7.71 ± 0.38 ^b^	7.29 ± 0.37 ^b^
Glucose	89.05 ± 1.44 ^a^	86.33 ± 1.61 ^a^	67.37 ± 1.69 ^c^	64.55 ± 1.99 ^c^
Myo-Inositol	-	-	1.00 ± 0.10 ^a^	1.10 ± 0.11 ^a^
2-deoxyglucose	-	-	-	-
Mannose				

Mean ± standard deviation (*n* = 3). Different letters in the same row indicate that values are significantly different (*p* > 0.05). MPE: mature peel; SMPE: semi-mature peel; MPU: mature pulp; SMPU: semi-mature pulp.

**Table 3:** Extractable and non-extractable phenolics content of umbu by-products (% bioaccessibility).

Sample	MPE	SMPE	MPU	SMPU
TEP	1229.43 ± 125.34 ^b^	1582.70 ± 278.09 ^a^	380.69 ± 7.76 ^d^	453.95 ± 40.29 ^c^
(mg GAE/100 g)	(15.67%)	(18.39%)	(32.92%)	(37.73%)
Tannins	1056.23 ± 55.20 ^b^	1350.9 ± 153.95 ^a^	110.64 ± 13.12 ^d^	170.14 ± 30.17 ^c^
(mg TAE/100 g)	(20.06%)	(18.81%)	(107.51%)	(114.27%)
Flavonoids	50.61 ± 1.90 ^b^	62.25 ± 3.60 ^a^	16.38 ± 2.36 ^d^	35.34 ± 3.11 ^c^
(mg QCE/100 g)	(39.10%)	(37.93%)	(34.39%)	(31.87%)
Phenolic acids (mg/100 g)	122.56	169.55	253.67	248.50
NEPA (mg/100 g)	901.89 ± 140.91 ^b^	1544.60 ± 296.58 ^a^	259.03 ± 12.50 ^c^	296.17 ± 13.09 ^c^
Hydrolyzable tannins (mg TAE/100 g)	1348.40 ± 19.86 ^a^	1079.70 ± 43.31 ^b^	655.91 ± 11.72 ^c^	535.88 ± 55.83 ^d^
Gallotannins (mg GAE/100 g)	139.89 ± 7.14 ^a^	124.97 ± 5.21 ^b^	63.73 ± 0.74 ^c^	55.66 ± 0.73 ^c^
Ellagitannins (mg EAE/100 g)	1208.51	954.73	592.18	480.22

Mean ± standard deviation (*n* = 3). Different letters in the same line indicate that values are significantly different (*p* > 0.05). MPE: mature peel; SMPE: semi-mature peel; MPU: mature pulp; SMPU: semi-mature pulp; TEP = Total extractable phenolics; NEPA = Non-extractable phenolics (proanthocyanidins).

**Table 4:** Phenolics contents of umbu pulps and peels flours.

Sample	*p*-Coumaric Acid (mg/100 g)	Protocatechuic Acid (mg/100 g)	Procyanidin B2 (mg/100 g)	Ellagic Acid (mg/100 g)	Quercetin (mg/100 g)
MPE	nd	nd	4.06 ± 0.33 ^b^	51.67 ± 4.67 ^b^	40.74 ± 9.26 ^b^
SMPE	9.16 ± 1.59 ^a^	nd	15.22 ± 0.97 ^a^	83.89 ± 6.53 ^a^	84.83 ± 6.41 ^a^
MPU	0.46 ± 0.16 ^c^	0.33 ± 0.03 ^b^	0.13 ± 0.02 ^d^	2.69 ± 0.49 ^c^	nd
SMPU	1.24 ± 0.45 ^b^	2.43 ± 0.03 ^a^	1.71 ± 0.13 ^c^	2.86 ± 0.93 ^c^	nd

Mean ± standard deviation (*n* = 3). Different letters in the same column indicate that values are significantly diferent (*p* > 0.05). MPE: mature peel; SMPE: semi-mature peel; MPU: mature pulp; SMPU: semi-mature pulp; nd: not detected.

**Table 5:** Carotenoids contents of umbu by-products for each analytical step.

Sample	MPE	SMPE	MPU	SMPU
Acetone extraction β-carotene (mg/100 g)	10.39 ± 0.23 ^b,x^	12.70 ± 0.80 ^a,x^	3.80 ± 0.20 ^c,x^	2.03 ± 0.04 ^d,x^
Partition β-carotene (mg/100 g)	5.90 ± 0.28 ^b,y^	6.52 ± 0.93 ^a, y^	1.91 ± 0.43 ^c,y^	1.06 ± 0.15 ^d,y^
Saponification β-carotene (mg/100 g)	2.28 ± 0.24 ^a,z^	1.40 ± 0.07 ^b, z^	0.51 ± 0.02 ^c,z^	0.13 ± 0.02 ^d,z^
Acetone extraction Zeaxanthin (mg/100 g)	11.52 ± 0.48 ^b^	14.64 ± 0.73 ^a^	4.78 ± 0.01 ^c,x^	2.19 ± 0.04 ^d,x^
Partition Zeaxanthin (mg/100 g)	6.64 ± 0.14 ^b,y^	7.25 ± 1.28 ^a,y^	22.06 ± 0.31 ^c,y^	1.18 ± 0.15 ^d,y^
Saponification Zeaxanthin (mg/100 g)	2.69 ± 0.10 ^a,z^	1.53 ± 0.09 ^b,z^	0.59 ± 0.03 ^c,z^	0.19 ± 0.07 ^d,z^

Mean ± standard deviation (*n* = 3). Different letters in the same column (a, b, c, d) and line (x, y, z) indicate that values are significantly different (*p* > 0.05). MPE: mature peel; SMPE: semi-mature peel; MPU: mature pulp; SMPU: semi-mature pulp.

**Table 6:** Carotenoids contents of umbu pulps and peels flours.

Sample	Lutein (µg/g)	α-Tocopherol (µg/g)	α-Carotene (µg/g)	Zeaxanthin (µg/g)	β-Cryptoxanthin (µg/g)	β-Carotene (µg/g)
MPE	0.58 ± 0.01 ^a^	3.40 ± 1.19 ^b^	2.04 ± 0.05 ^a^	0.46 ± 0.01 ^a^	5.49 ± 0.36 ^a^	5.03 ± 0.04 ^a^
SMPE	0.62 ± 0.06 ^a^	6.86 ± 0.44 ^a^	1.71 ± 0.18 ^b^	0.20 ± 0.00 ^c^	2.30 ± 0.11 ^b^	3.30 ± 0.17 ^b^
MPU	0.59 ± 0.01 ^a^	1.79 ± 0.09 ^c^	0.37 ± 0.03 ^c^	0.22 ± 0.00 ^b^	1.79 ± 0.15 ^c^	1.11 ± 0.05 ^c^
SMPU	0.50 ± 0.01 ^b^	1.83 ± 0.08 ^c^	0.10 ± 0.03 ^d^	0.15 ± 0.01 ^d^	0.82 ± 0.02 ^d^	0.97 ± 0.04 ^d^

Mean ± standard deviation (*n* = 3). Different letters in the same column indicate that values are significantly different (*p* > 0.05). MPE: mature peel; SMPE: semi-mature peel; MPU: mature pulp; SMPU: semi-mature pulp.

**Table 7:** Proximal composition of umbu peels flours.

Composition (g/100 g)					
Sample	Moisture	Fat	Ash	Protein	Carbohydrate
MPE	8.16 ± 0.14 ^b^	0.69 ± 0.28 ^a^	3.40 ± 0.21 ^a^	5.87 ± 0.75 ^a^	81.91
SMPE	7.56 ± 0.18 ^a^	0.71 ± 0.31 ^a^	2.61 ± 0.32 ^b^	4.49 ± 0.75 ^a^	84.63

Average value ± standard deviation. Different letters in the same column indicate that values are significantly different (*p* > 0.05). MPE: mature peel; SMPE: semi-mature peel.

**Table 8:** Technological properties of umbu peels flours.

Sample	L*	H	C*	WRC (g water/g)	ORC (g oil/g ms)	SWC (ml/g ms)	WSI (g/100 g)
MPE	62.49 ± 0.13 ^b^	78.58 ± 0.08 ^b^	32.91 ± 0.29 ^a^	3.74 ± 0.32 ^a^	1.77 ± 0.24 ^a^	6.35 ± 1.01 ^a^	20.42 ± 1.81 ^a^
SMPE	64.69 ± 0.20 ^a^	80.03 ± 0.13 ^a^	28.18 ± 0.05 ^b^	3.95 ± 0.12 ^a^	1.64 ± 0.02 ^a^	5.87 ± 0.56 ^a^	14.34 ± 0.74 ^b^

Average value ± standard deviation. Different letters in the same column indicate that values are significantly different (*p* > 0.05). MPE: mature peel; SMPE: semi-mature peel.

## Data Availability

The data presented in this study are available on request from the corresponding author. The data are not publicly available at this moment because they are part of an ongoing PhD Thesis.
